# Establishment of Green Fluorescent Protein and Firefly Luciferase Expressing Mouse Primary Macrophages for In Vivo Bioluminescence Imaging

**DOI:** 10.1371/journal.pone.0142736

**Published:** 2015-11-10

**Authors:** Jukka Pajarinen, Tzu-hua Lin, Taishi Sato, Florence Loi, Zhenyu Yao, Yrjö T. Konttinen, Stuart B. Goodman

**Affiliations:** 1 Orthopaedic Research Laboratories, Department of Orthopaedic Surgery, Stanford University School of Medicine, Stanford, CA, United States of America; 2 Department of Medicine, Institute of Clinical Medicine, University of Helsinki, Helsinki, Finland; Wayne State University, UNITED STATES

## Abstract

Macrophages play a key role in tissue homeostasis as well as in a range of pathological conditions including atherosclerosis, cancer, and autoimmunity. Many aspects of their in vivo behavior are, however, poorly understood. Bioluminescence imaging (BLI) with green fluorescent protein (GFP) and firefly luciferase (FLUC) labelled autologous reporter macrophages could potentially offer a powerful tool to study macrophage biology, but this approach has been hindered by the relative difficulty of efficient gene transfer into primary macrophages. Here we describe a straightforward method for producing large numbers of GFP/FLUC expressing mouse primary macrophages utilizing lentivirus vector, cyclosporine, and a double infection strategy. Using this method we achieved up to 60% of macrophages to express GFP with correspondingly high FLUC signal. When injected into the circulation using a mouse model of local biomaterial induced inflammation and osteolysis, macrophages were initially detectable within the lungs, followed by systemic homing to the local area of chronic inflammation in the distal femur. In addition, transduced macrophages maintained their ability to assume M1 and M2 phenotypes although the GFP/FLUC expression was altered by the polarizing signals. These reporter macrophages could prove to be valuable tools to study the role of macrophages in health and disease.

## Introduction

Macrophages play a key role in tissue homeostasis as well as in a wide range of pathological conditions including atherosclerosis, cancer, and autoimmunity [1, 2]. Due to their critical role as regulators of acute and chronic inflammation as well as tissue regeneration, macrophages have attracted growing interest also in such fields as regenerative medicine and biomaterial science [3–5]. In all these fields of biomedicine, green fluorescent protein (GFP) and firefly luciferase (FLUC) expressing mouse primary macrophages could potentially be valuable tools for cell trafficking studies done using in vivo bioluminescence imaging. However, production of such reporter cells possesses a technical challenge due to the relative difficulty of efficient gene transfer into primary macrophages [6]. Following the initial description of cyclosporine as an effective facilitator of lentivirus mediated gene transfer into mouse primary cells [6], we describe here an effective method for production of GFP and FLUC expressing primary mouse macrophages for various *in vivo* bioluminescence imaging and cell trafficking studies using cyclosporine and lentivirus mediated gene transfer.

## Materials and Methods

### Lentivirus production

Human embryonic kidney 293T cells (ATCC, Manassas, VA) were cultured in Dulbecco’s modified eagle medium (Life Technologies, Pleasanton, CA) supplied with 10% heat inactivated MSC grade fetal bovine serum (FBS, Invitrogen, Waltham, MA) and antibiotic-antimycotic solution (100 units of penicillin, 100μg of streptomycin, and 0.25 μg of Amphotericin B per ml; Hyclone, Thermo Scientific, Waltham, MA). Human immunodeficiency virus-1 based vesicular stomatitis virus-G (VSV-G) pseudotype lentivirus particles were generated by co-transfecting the pFU-Luc2-eGFP lentivirus vector, psPAX2 packaging vector, and pMD2G VSV-G envelope vector into 293T cells using calcium phosphate transfection kit (Clontech, Mountain View, CA) with 25μM chloroquine. The culture supernatant was collected 48 h post-transfection and the cellular debris was removed by centrifugation. The virus titer was determined by using 293T cells; the titer of multiplicity of infection (MOI) on 293T cells were used to calculate the virus amount used in macrophage infection. The supernatant was stored in -80°C until used.

### Isolation and culture of mouse bone marrow macrophages

Mouse bone marrow macrophages (mBMMs) were collected from the femora and tibiae of 40 male BALB/cByJ mice (The Jackson Laboratory, Bar Harbor, ME) aged 6 to 8 weeks using established protocols [7, 8]. For comparison mBMMs were isolated also from one male, 5-weeks-old, L2G85 BALB/c mouse harboring CAG-luc-eGFP L2G85 transgene (The Jackson Laboratory, stock number 010545). The mice were euthanized with CO_2_ followed by cervical dislocation, sterilized in 70% ethanol for 2 x 5 min after which femora and tibiae were collected under aseptic conditions. The proximal and distal ends of the bones were transversally cut and the bone marrow was flushed out by injecting 5 ml of Gibco RPMI 1640 (Life Technologies) medium supplemented with 10% heat inactivated Gibco FBS (Life Technologies) and 1% Gibco Antibiotic- Antimycotic (Life Technologies) into the medullary canal with a syringe and 25-gauge needle. The cells were filtered through a 100 μm cell strainer and centrifuged. Cells were suspended in ice cold red blood cell lysis buffer (Sigma-Aldrich, St. Louis, MO) and after 2 min media was added to stop the cell lysis. Cells were centrifuged again and resuspended in complete mBMM culture media comprising of RPMI 1640 with 10% FBS, 30% L929 cell-conditioned medium, 10 ng/mL macrophage colony-stimulating factor (M-CSF, R&D Systems, Minneapolis, MN) and 1% Gibco Antibiotic- Antimycotic (Life Technologies). Cells were counted and plated into 175 cm^2^ culture flasks (BD, Franklin Lakes, NJ), 5 x 10^7^ cells per flask. Cells were cultured for 6 days during which the media was changed on days 2 and 4 to remove non-adherent cells. After reaching confluence cells were lifted with Gibco 0.25% Trypsin-EDTA (Life Technologies) and gentle scraping, centrifuged and split 1:2 either into 175 cm^2^ culture flasks or 10 cm diameter cell culture dishes. After subculture, mBMMs were allowed to recover and expand for 5 days and reach 50 to 70% confluence prior to lentivirus infection.

### mBMMs transduction

The supernatant from transfected 293T cells was mixed with complete mBMM culture media at an approximate ratio of 2:1 and was further supplemented with 6 μg/ml polybrene (Sigma-Aldrich), 10 μg/ml sterile filtered DEAE-dextran (Sigma-Aldrich) or 10 μM cyclosporine (Sigma-Aldrich). To achieve a MOI of 10, 6 ml of this infection media was used for macrophages in 10 cm dishes and 15 ml for the cells in 175 cm^2^ culture flasks. After 24 hours infection media was removed and complete mBMM media added. The cells were allowed to recover for 24 hours after which the infection was repeated in selected groups (single or double infection). Three days after the second infection the GFP expression was determined by fluorescence microscopy and flow cytometry. The FLUC activity was determined using a Luciferase Assay System kit (Promega, Madison, WI) and a Turner BioSystems Luminometer TD-20/20 (Promega). The FLUC activity was normalized to the amount of total protein measured using a Pierce BCA Protein Assay Kit (Thermo Scientific). The viability of the reporter macrophages was assessed three days after the second infection by flow cytometry and propidium iodide staining

### Reporter macrophage polarization

Three days after the second infection, GFP and FLUC expressing reporter macrophages transduced with cyclosporine and lentivirus as well as non- transduced control macrophages were treated either with 100 ng/ml lipopolysaccharide (LPS, Escherichia coli 0127:B8, Sigma-Aldrich), 10 ng/ml LPS with 20 ng/ml interferon gamma (IFN-γ, R&D systems), 20 ng/ml IFN-γ or 20 ng/ml IL-4 (R&D systems) for 24 hours. After the polarization the cells were lysed for total RNA extraction with lysis buffer of the RNeasy kit (Qiagen, Venlo, Limburg, Netherlands) or cultured 2 more days after which GFP expression and FLUC activity were determined.

### qRT PCR

Total RNA was extracted using an RNeasy Mini kit following the manufacturer’s instructions. The quality and the amount of the total RNA were measured with a NanoDrop 1000 spectrophotometer (Thermo scientific). Complementary DNA (cDNA) was synthesized from an equal amount of total RNA from each sample using a high-capacity cDNA archive kit (Applied Biosystems, Foster City, CA) and a thermocycler (Eppendorf, Hamburg, Germany). For quantitative real-time PCR, a reaction mix containing sample cDNA, one of 18S, TNF-α, iNOS, IRF5, CD206, Arg1 or IRF4 TaqMan primer-probes and TaqMan Gene Expression Master Mix (all from Applied Biosystems) was prepared. qRT-PCR was performed with ABI 7900HT Sequencing Detection System (Applied Biosystems) using 18S rRNA as an internal control. The results were obtained by use of the comparative Ct method [9].

### Mouse model of wear debris induced inflammation and osteolysis

The *in vivo* performance of GFP and FLUC expressing reporter macrophages was assessed in a mouse model of biomaterial wear debris induced local inflammation and osteolysis [10–12]. The study was carried out in strict accordance with the recommendations in the Guide for the Care and Use of Laboratory Animals of the National Institutes of Health. The protocol was approved by the Administrative Panel on Laboratory Animal Care (APLAC) at Stanford University (Protocol Number: 17566). Briefly, Alzet model 2006 miniature osmotic pumps (Durect Corporation, Cupertino, CA) were loaded with ultra-high-molecular-weight polyethylene particles (mean diameter 0.48 ± 0.10 μm) suspended in 1% BSA-PBS at a concentration of 15 mg/ml. Pumps were connected to hollow titanium rods (0.82 mm x 6 mm) via 6 cm long vinyl tubing prefilled with the particle solution. A skin incision was made to the lateral side of the right hip or flank region of 10, male, 8 to 12 weeks old BALB/cByJ mice under isoflurane anesthesia. The joint capsule of the right knee was opened by a lateral parapatellar arthrotomy to access the intercondylar notch of the distal femur after which a series of needles was used to drill through the notch to gain access to the medullary cavity. The pump with the tubing was then implanted in the subcutaneous tissues in the back of the mice via a second skin incision made to the dorsal side of the neck. A subcutaneous tunnel reaching the right knee was made for the tubing. The titanium rod, connected from the one end to the pump via tubing, was then press fit into the drill hole in right distal femur resulting in a continuous delivery of UHMWPE particles to the medullary cavity with accompanied chronic inflammation and local osteolysis. Skin incisions were closed with 5–0 Ethilon sutures. Repeated s.c. buprenorphine injections were used in the postoperative analgesia.

### In vivo bioluminescence imaging

Seven days after the operation, 6x10^6^ M0 reporter macrophages suspended in 150μl of HBSS were injected into the tail vein of the mice and the systemic trafficking of the reporter cells was observed by obtaining lateral and prone BLI images of the mice immediately after the cell injection and then at 2 day intervals up to 20 days post injection using IVIS 100 system (Xenogen, Alameda, CA). Ten minutes prior to imaging mice received 3 μg intraperitoneal injection of D-luciferin (Biosynth, Staad, Switzerland). The BLI images were quantified by measuring the total flux (photons/second) from uniformly sized regions of interest (ROI) drawn over the right and left distal femur using Living Image (V 4.1. Perking Elmer, Waltham, MA) image analysis software.

### Immunohistochemical detection of reporter macrophages

21 days after the systemic injection of reporter macrophages, the mice were euthanized with CO_2_ followed by cervical dislocation. The right and left femurs of three representative mice were dissected free of soft tissues, fixed in 4% paraformaldehyde for 3 days, decalcified in 0.5M ethylenediaminetetraacetic acid PBS for 2 weeks and then embedded in OCT compound for sectioning. The distal metaphyseal region of the right femur representing the area of biomaterial inflammation and the corresponding anatomical area of left control femur were sectioned into 8μm thick frozen sections in the transverse plane and the reporter macrophages detected in three 10 to 20 μm spaced sections by firefly luciferase immunostaining. For the immunostaining tissue sections were permeabilized in 0.1% Triton-X PBS for 15 min and washed in PBS 3x5min. Endogenous peroxidase activity in the sections was blocked by 10 minute treatment in Bloxall (Vector Laboratories, Burlingame, CA), followed by a wash in PBS for 5 min, and blocking in 10% goat normal serum (Vector Laboratories) containing added Avidin from Avidin/Biotin Blocking Kit (Vector Laboratories) in 1% BSA-PBS for 1 hour at room temperature. Normal serum was then decanted, sections briefly rinsed, and polyclonal rabbit anti-firefly luciferase antibody, 5μg/ml, diluted in 1% BSA-PBS (ab21176, Abcam, Cambridge, MA) applied to the sections with added biotin from the Avidin/Biotin Blocking Kit. After an overnight incubation at +4°C the sections were washed 3x5min in PBS and incubated in biotinylated goat anti-rabbit secondary antibody (Vector Laboratories) diluted 1/200 in 1% BSA-PBS for 1 hour at room temperature. Sections were washed again 3x5min in PBS and incubated with AB complex from Vectastain ABC kit (Vector Laboratories) for 1 hour at room temperature. After 3x5min washes in PBS the sections were treated with ImmPACT NovaRED peroxidase substrate (Vector Laboratories) for 10 min to visualize the sites of antibody binding and counterstained with hematoxylin. Sections were dehydrated in graded ethanol series, cleared in xylene and mounted with VectaMount (Vector Laboratories). The amount of positive cells was compared between the left and right femurs. Specify of the immunostaining was confirmed by including negative staining controls performed without the addition of the primary antibody. The specificity of the immunostaining was further confirmed by staining GFP-FLUC transduced (positive control) and non-transduced (negative control) RAW 264.7 cells (TIB-71, ATCC, Manassas, VA) using the same fixation and firefly luciferase immunstaining protocol as for the tissue sections.

### Statistical analyses

All data is presented as mean ± standard error of the mean derived from three independent experiments. When appropriate, student t-test was used for pair-wise comparison of variables and one-way ANOVA with post hoc Tukeys’ multiple comparison test for the group analyses. Pearson correlation coefficient was used to analyze correlation between the variables. Two-sided p values less than 0.05 were considered significant. Statistical analyses were conducted using Graphpad Prism version 6.03 (GraphPad Software, La Jolla, CA).

## Results

### Cyclosporine was superior to polybrene and dextran in facilitating macrophage transduction

**Fig 1:**
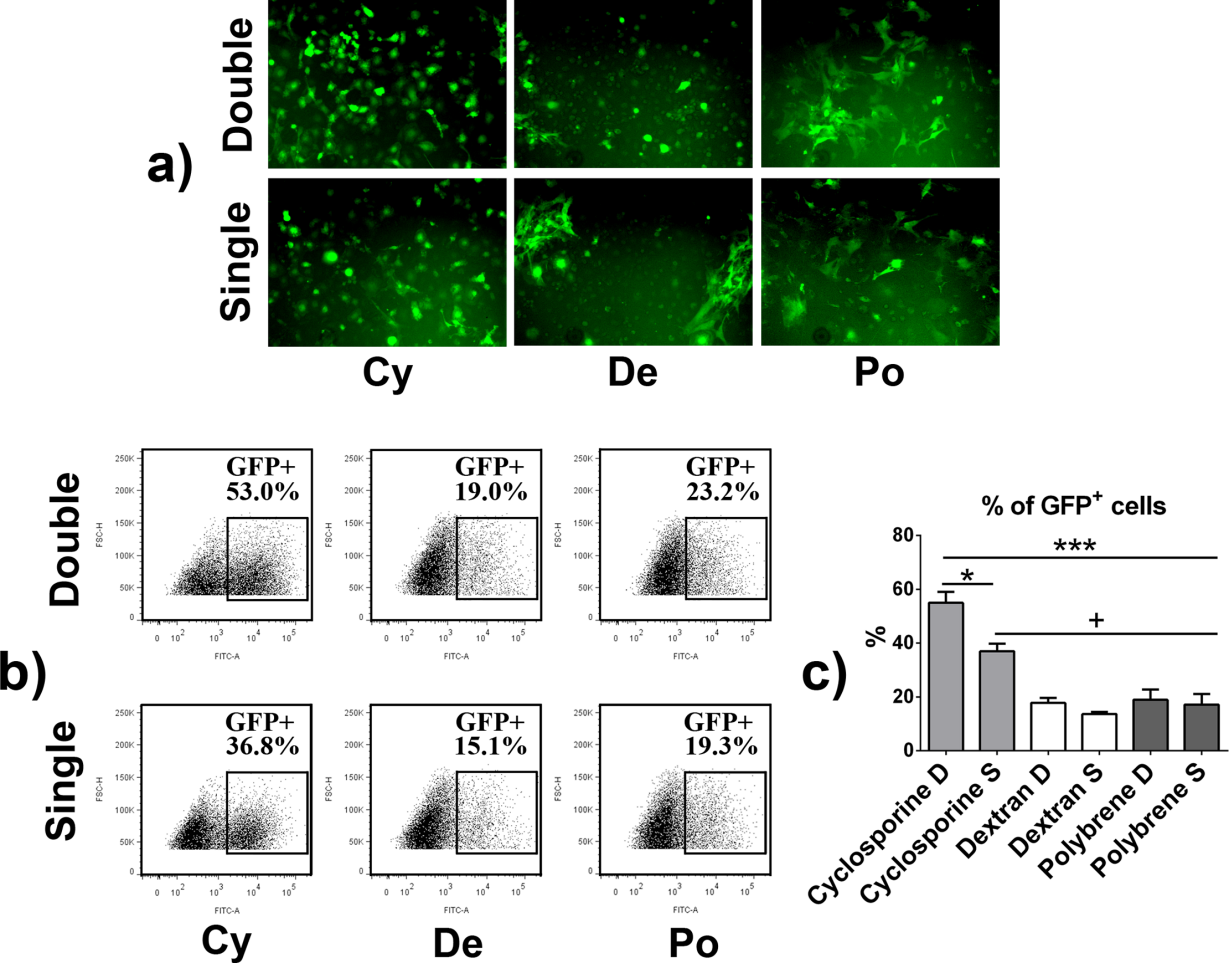
GFP expression in reporter macrophages. GFP and FLUC expressing reporter macrophages were produced by transducing mouse bone marrow macrophages with lentivirus vector in the presence of cyclosporine, DEAE-dextran or polybrene. The 24-hour virus infection was done either once or repeated after a 24-hour rest period (single vs. double infection). **a)** Fluorescence microscopy images of the GFP positive macrophages in the indicated treatment groups three days after the second infection. **b)** Representative scatter blots of GFP positive reporter macrophage in flow cytometry. **c)** Percentage of GFP positive macrophages in the indicated treatment groups as determined by flow cytometry. Cy–Cyclosporine; De–DEAE-dextran; Po–Polybrene; D–Double infection; S–Single infection; *** = p > 0.001 cyclosporine double infection vs. dextran and polybrene single and double infection; * = p > 0.05 cyclosporine double vs. single infection; + = p > 0.05 cyclosporine single infection vs. dextran and polybrene single and double infection.

After a single 24-hour infection with lentivirus in the presence of polybrene or dextran, 17.1 ± 3.9% or 13.6 ± 0.8%, of the macrophage population expressed GFP as assessed by fluorescent microscopy and flow cytometry. Polybrene and dextran were equally effective in facilitating the gene transfer with no significant differences observed in the transduction efficiency. In contrast, significantly higher GFP expression (37.0 ± 2.8%, p < 0.05) was observed after cyclosporine mediated lentivirus infection (**Fig 1A, 1B and 1C**).

### Repeating the lentivirus infection enhanced the transduction efficiency

**Fig 2:**
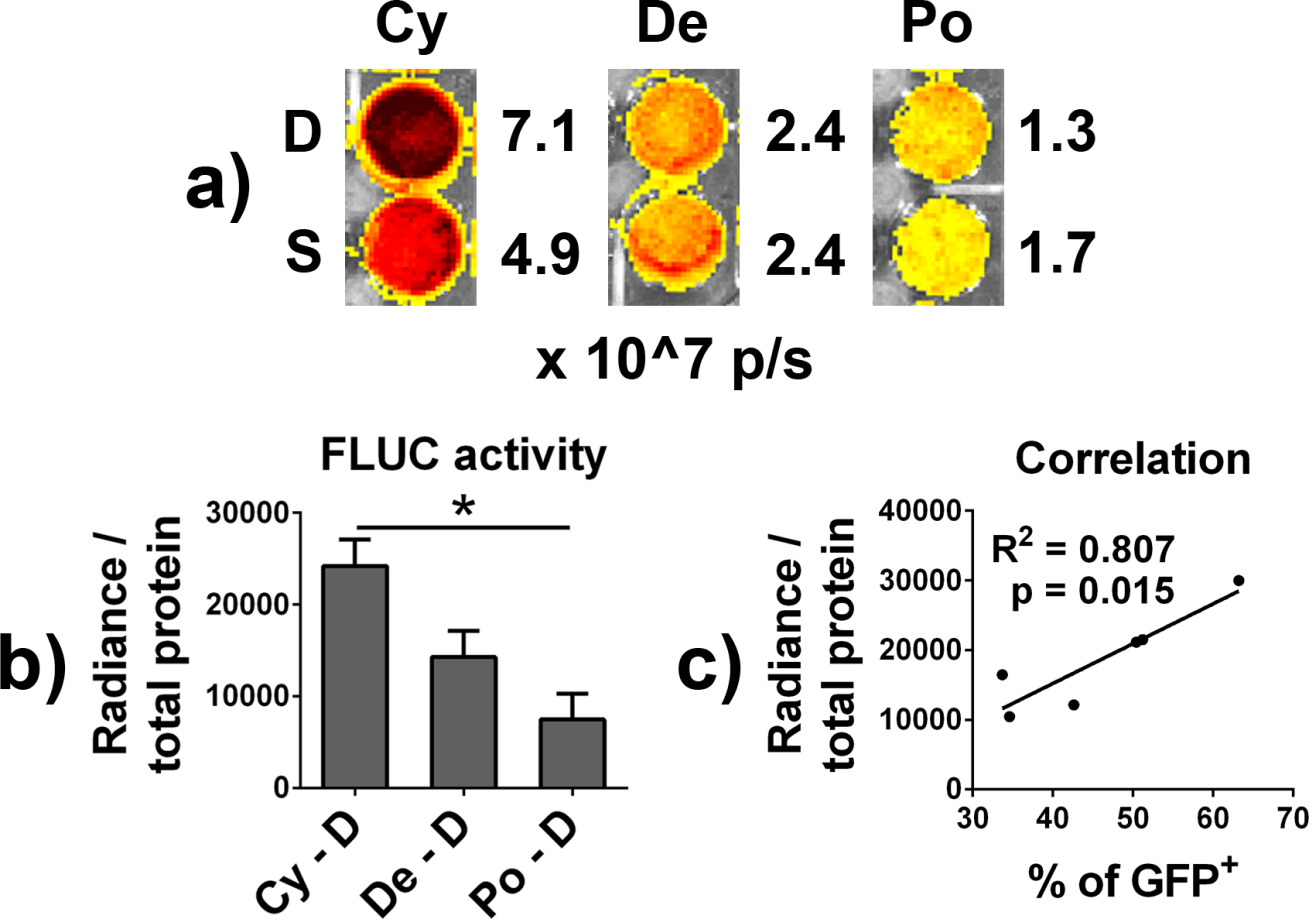
FLUC expression in reporter macrophages. GFP and FLUC expressing reporter macrophages were produced by transducing mouse bone marrow macrophages with lentivirus vector in the presence of cyclosporine, DEAE-dextran or polybrene. The 24-hour virus infection was done either once or repeated after a 24-hour rest period (single vs. double infection). **a)** Reporter macrophages from the indicated treatment groups were seeded to 24-well plate at standardized density three days after the second infection. D-luciferin was added to the wells and immediately after luminescence signal emanating from each well imaged using IVIS 100 system and quantified with Living Image software. **b)** Luciferase activity in indicated double infected reporter macrophage lysates as determined by Luciferase Assay System kit and luminometer. **c)** Person correlation between the amount of GFP positive cells and normalized FLUC activity in macrophages infected once or twice in the presence of cyclosporine. D–Double infection; S–Single infection; Cy–Cyclosporine; De–DEAE-dextran; Po–Polybrene. * = p > 0.05 cyclosporine double infection vs. polybrene double infection.

Repeating the lentivirus infection further increased the transduction efficiency in the presence of cyclosporine (54.9 ± 4.1%, p < 0.05 vs. single infection with cyclosporine; p < 0.001 vs. single or double infection with polybrene or dextran) but not when polybrene or dextran facilitated the gene transfer (19.0 ± 3.8% or 17.8 ± 1.9%, correspondingly) (**Fig 1A, 1B and 1C**). Normalized FLUC activity in the reporter cells closely followed the pattern of GFP expression; double infected cyclosporine group showed the strongest FLUC signal (2.4 ± 0.29 x10^4^ in cyclosporine treated group vs. 0.7 ± 0.28 x10^4^ in polybrene group vs. 1.4 ± 0.29 x10^4^ in dextran group) with the difference between cyclosporine and polybrene treated groups reaching statistical significance (**Fig 2A, and 2B**). A strong positive correlation between the number of GFP positive cells and normalized FLUC activity was observer (Pearson correlation coefficient 0.90, p = 0.015) (**Fig 2C**). No differences in the cell viability were observed between the treatment groups (**Table 1**).

**Table 1:** Reporter macrophage viability. GFP and FLUC expressing reporter macrophages were produced by transducing mouse bone marrow macrophages with lentivirus vector in the presence of cyclosporine, DEAE-dextran or polybrene. The 24-hour virus infection was done either once or repeated after a 24-hour rest period (single vs. double infection). The percentage of viable cells was assessed three days after the second infection by flow cytometry and propidium iodide staining.

Treatment group	Viability (%)	
	Single infection	Double infection
Cyclosporine	96.6	96.3
Dextran	97.5	97.1
Polybrene	96.4	97.1

### Transduced macrophages showed stronger GFP/FLUC expression than cells from the transgenic mouse

Only small portion of mBMMs isolated from the transgenic L2G85 BALB/c mouse expressed GFP (1.07% in L2G85 macrophages vs. 54.9% in cyclosporine double infection group) with correspondingly weak FLUC activity (0.02 x10^4^ in L2G85 macrophages vs. 2.4 x10^4^ in cyclosporine double infection group).

### Gene transfer did not impact macrophages ability to assume M1 and M2 phenotypes

**Fig 3:**
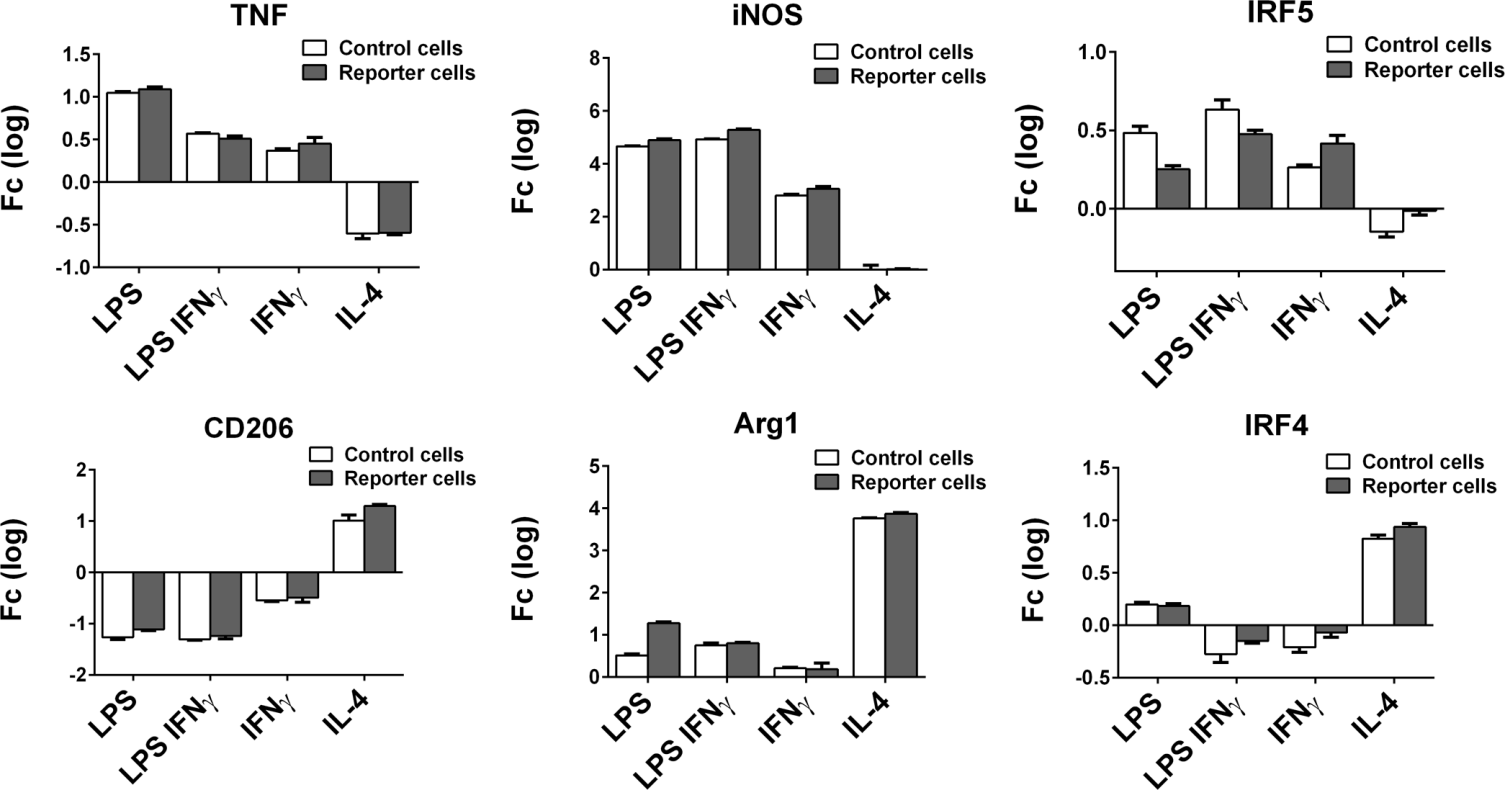
Reporter macrophage polarization. Lentivirus and cyclosporine transduced reporter macrophages and non-transduced control macrophages were polarized into M1 and M2 phenotypes three days after the virus infection and the expression of M1-related genes TNF-α, iNOS and IRF5 as well as M2-realted genes CD206, Arg1 and IRF4 was determined by qRT PCR. Results are expressed as common logarithm-transformed fold change (fc) to corresponding non-polarized M0 macrophages.

Non-transduced control macrophages and GFP/FLUC expressing reporter macrophages, transduced with lentivirus and cyclosporine, upregulated TNF-α, iNOS and IRF5 mRNA as a response to LPS, LPS+IFNγ or IFNγ treatment in nearly identical manner (**Fig 3**). Likewise, IL-4 treatment led to down regulation of TNF-α and upregulation of CD206, Arg1 and IRF4 mRNA in both control macrophages and transduced macrophages, with no significant differences being detected between the groups in the expression of these M1 and M2 related marker genes (**Fig 3**).

### GFP/FLUC expression was decreased by M1 and increased by M2 polarization

**Fig 4:**
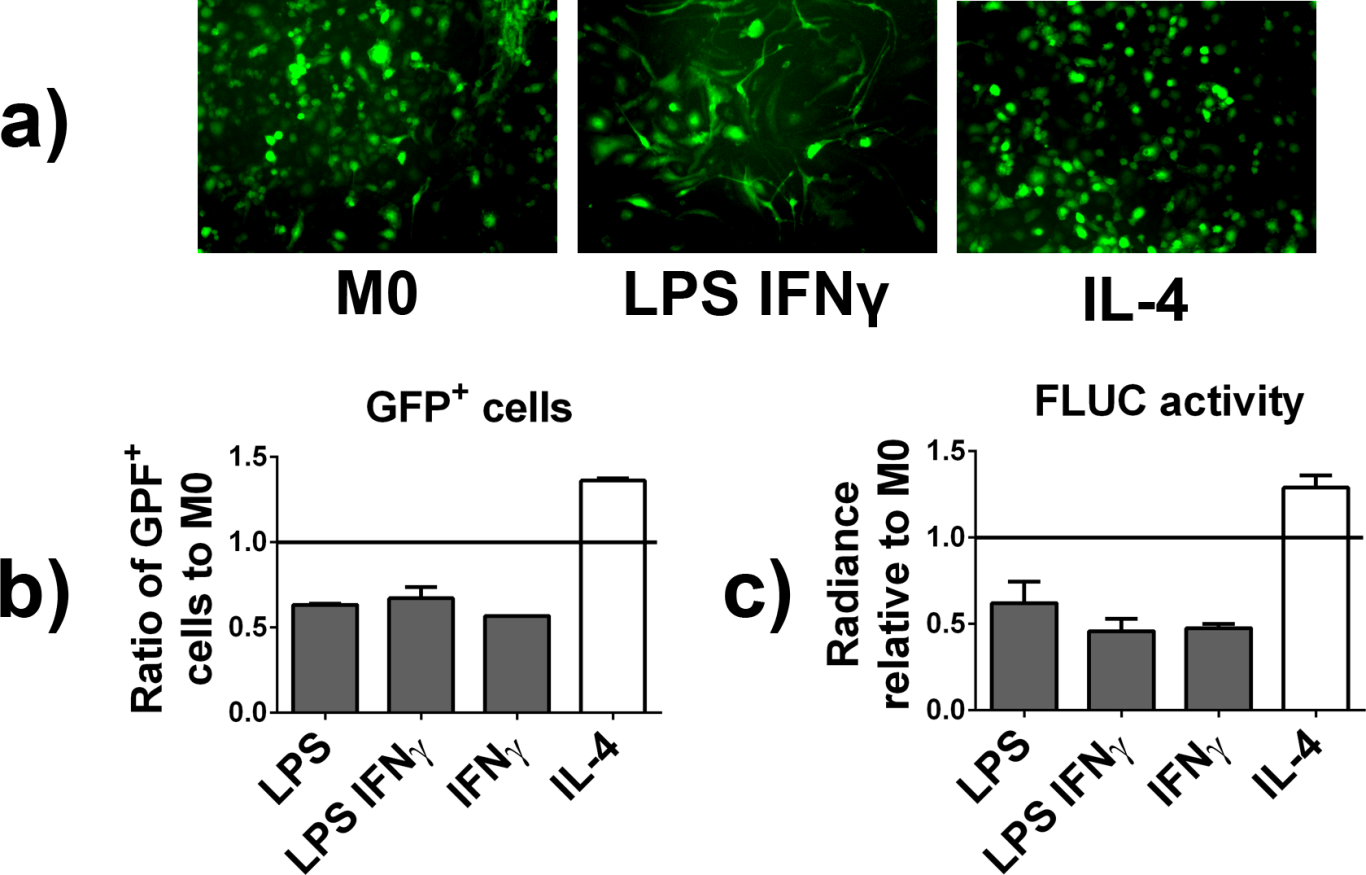
GFP and FLUC expression in polarized reporter macrophages. Lentivirus and cyclosporine transduced reporter macrophages were polarized into M1 and M2 phenotypes or left untreated (M0 macrophages) and the expression of GFP and FLUC determined 48 hours after the end of the polarization treatment. **a)** Fluorescence microscopy images of the GFP positive macrophages in the indicated treatment groups. **b)** Ratio of GFP positive macrophages in the indicated treatment groups compared to non-polarized M0 reporter macrophages as determined by flow cytometry **c)** FLUC activity in indicated treatments groups compared to the non-polarized M0 reporter macrophages as determined by Luciferase Assay System kit and luminometer.

Treatment of the GFP/FLUC expressing reporter macrophages with LPS, LPS+IFNγ or IFNγ led into 36.8 ± 0.01%, 33.0 ± 0.05% or 43.0 ± 0.00% reduction in GFP expression with closely corresponding reduction in FLUC activity (38.0 ± 0.12%, 54.3 ± 0.1% and 52.5 ± 0.02%) as compared to non-treated M0 macrophages (**Fig 4A, 4B and 4C**). In contrast, IL-4 treatment led to 36.1 ± 0.01% increased GFP expression with corresponding 28.8 ± 0.07% increase in FLUC activity as compared to non-polarized M0 macrophages (**Fig 4A, 4B and 4C**). Next we performed transcription factor binding element analysis of the ubiquitin promoter driving the expression of GFP/FLUC using TFSEARCH [13]. We identified a total of 88 high-scoring (>85.00) transcription factor biding sites, including a binding site for M2-associated transcription factor CREB. A full list of the identified transcription factor binding sites is given as supplementary information (**S1 Table**).

### Reporter macrophages migrated to the local site of chronic inflammation in vivo

**Fig 5:**
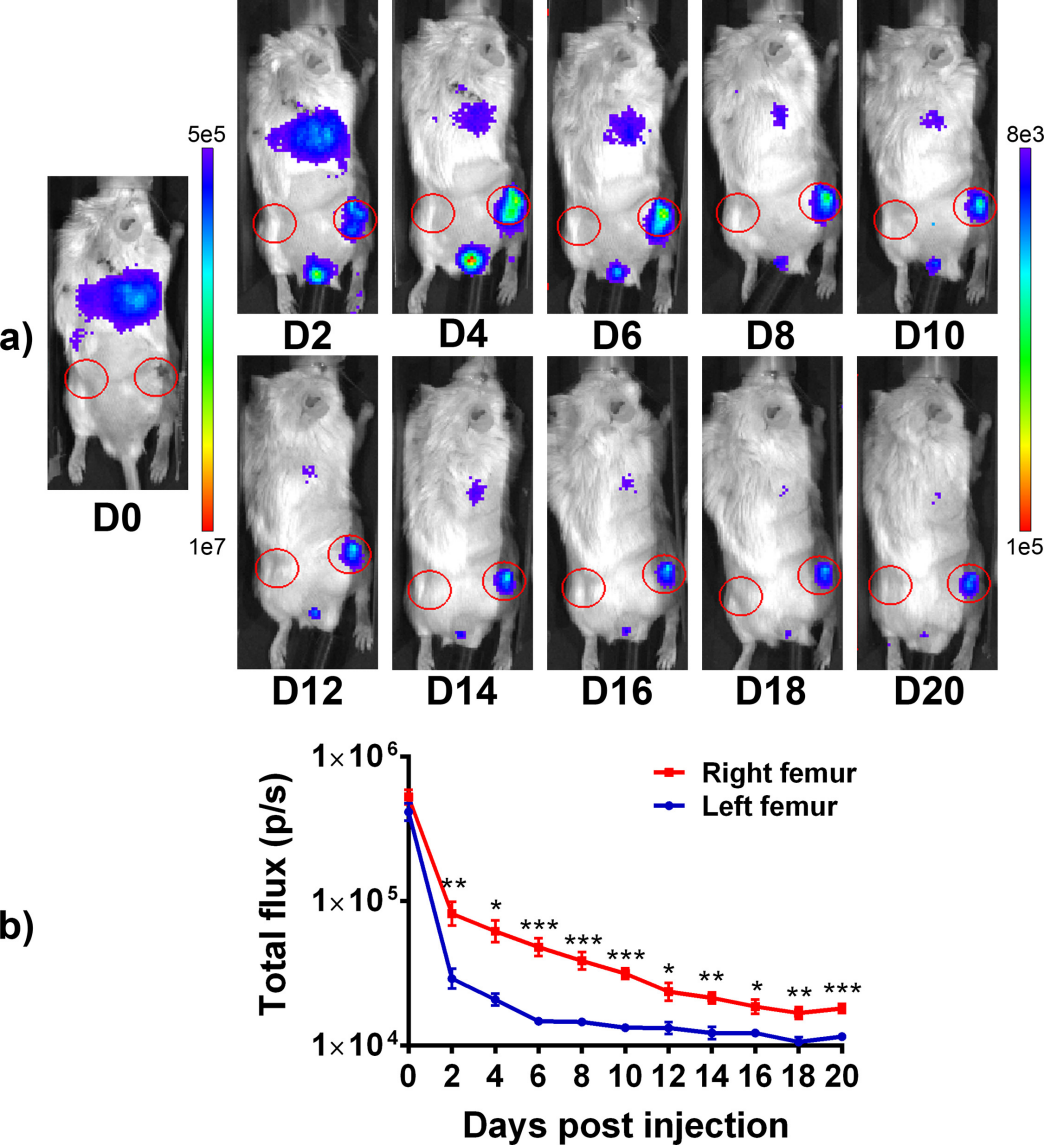
Reporter macrophages in in vivo bioluminescence imaging. Lentivirus and cyclosporine transduced reporter macrophages where injected into the tail vein of mouse model of biomaterial induced local inflammation and osteolysis. The cell trafficking was followed by bioluminescence imaging. Luminescence emanating from the local area of inflammation in the right distal femur as well as left distal femur serving as a control was determined from images obtained every other day up to 20 days post injection. **a)** Bioluminescence images showing the accumulation of reporter cells to the right distal femur starting on the day 2 after the systemic injection of the reporter cells and persisting over the 20 day imaging period. **b)** XY-blot showing the total flux from regions of interest over the right and left distal femurs. * = p > 0.05; ** = p > 0.01, *** = p > 0.001.

**Fig 6:**
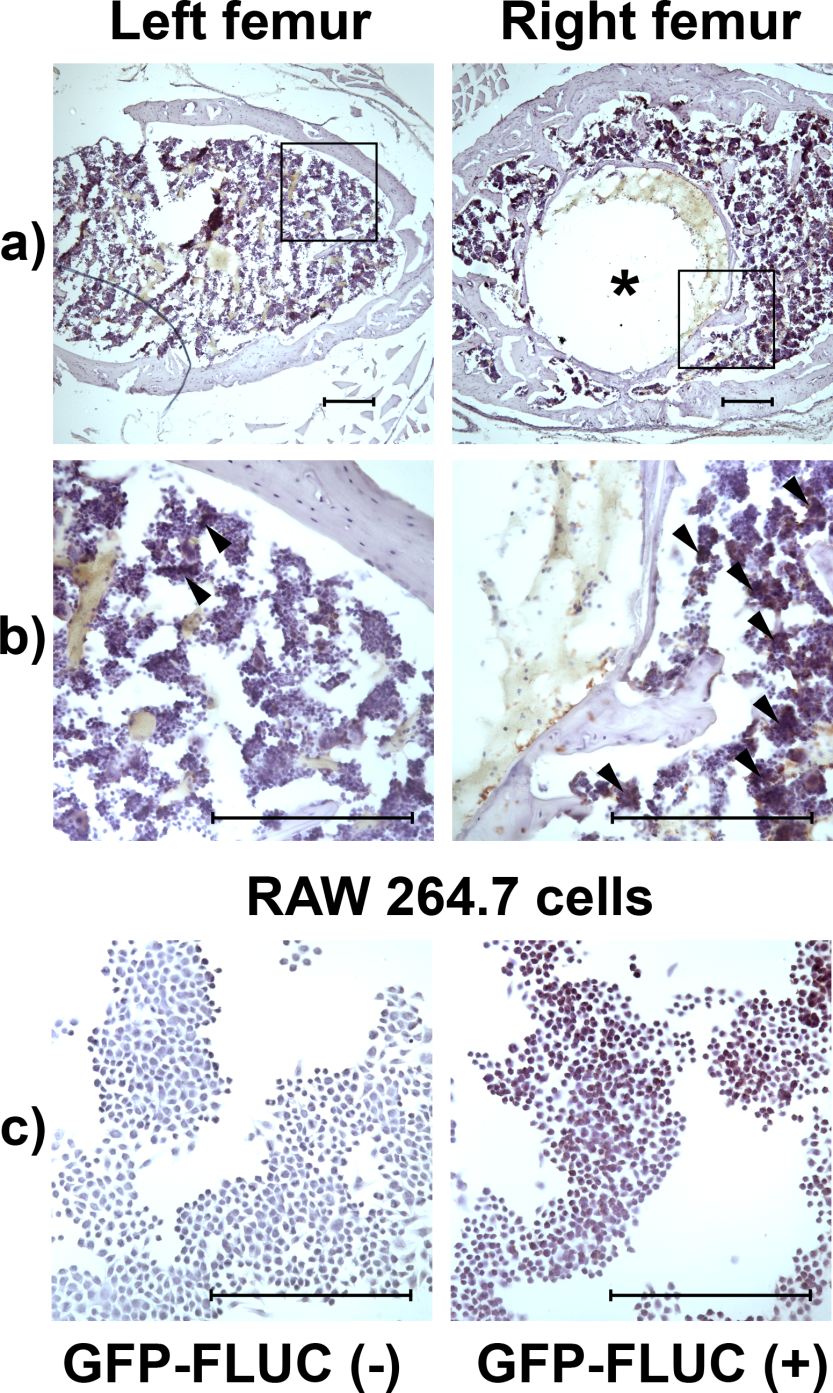
Detection of reporter macrophages from tissue sections. Lentivirus and cyclosporine transduced reporter macrophages where injected into the tail vein of mouse model of biomaterial induced local inflammation and osteolysis. Reporter cell accumulation to the right distal femur was confirmed by bioluminescence imaging. After 21 days the right and left femur were collected, processed into frozen sections and the reporter macrophages detected by FLUC immunostaining. **a)** Low power micrographs showing the transverse sections of distal metaphyseal region of left and right femur. On the right side the channel left behind by the titanium rod is evident (asterisk). **b)** High power micrographs showing several aggregations of FLUC positive cells (arrow heads) in the bone marrow surrounding the rod channel on the right side while only few FLUC positive cells are detected in the bone marrow of the left femur. **c)** GFP-FLUC transduced and non-transduced RAW 264.7 cells stained for FLUC served as additional positive and negative immunostaining controls. Scale bars 200μm.

Immediately after injection into the mouse circulation, a strong BLI signal emanating from the area corresponding to the lungs was detected. No difference in the signal from right and left femur was observed at this stage, although the initial strong signal from the lungs was reflected to these regions of interest as evident from very high initial flux from both of these ROIs (left 4.1 ± 1.8 x10^5^ vs. right 5.5 ± 1.7 x10^5^ p/s, not significant). Starting from day 2 post-injection, BLI signal developed over the right distal femur significantly exceeding the signal from the left femur. Although steadily decreasing, the significantly increased signal from the right femur compared to the left femur persisted over the 20-day imaging period (**Fig 5A and 5B**). In histological sections of left and right femurs obtained at day 21 after the cell injection and stained for FLUC, clear accumulations of FLUC positive cells were evident in the bone marrow surrounding the channel left behind by the implanted titanium rod while only few such cells were seen on the left femur (**Fig 6A and 6B**). No non-specific staining was observed in the negative staining controls. The GFP-FLUC transduced RAW 264.7 cells stained for FLUC showed positive signal while no signal was detected in the non-transduced cells further confirming the specificity of the FLUC immunostaining (**Fig 6C**).

## Discussion

Macrophages are key regulators of acute and chronic inflammation as well as tissue regeneration, and have been recognized to play a role in wide variety of pathological conditions such as cancer, infection and others [1–4]. Despite the considerable advances made in the understanding of macrophage biology, e.g. the description of macrophage polarization, many of their characteristics still remain to be elucidated. In particular, macrophage activation, function, migration and survival in the in vivo setting are poorly understood. In vivo bioluminescence imaging with GFP/FLUC labelled autologous reporter macrophages could potentially offer a powerful tool to study these poorly characterized aspects of macrophage biology.

Indeed, some approaches to this end have already been developed. GFP/FLUC expressing mouse macrophage cell lines are relatively easy to produce using standard transduction methods and have been used to study macrophage trafficking in immunocompromised mice [11, 12]. However, the use of cell lines for this application has limitations including the non-autologous cell transfer necessitating the use of immunocompromised mice, the malignant nature of the cells eventually resulting in tumor formation leukemia and, presumably, less physiological behavior of cell lines compared to primary cells. Production of FLUC transgenic mouse models followed by isolation of primary macrophages from these mice for autologous reporter cell transfer is a potentially powerful approach to the problem. Indeed, high expression of GFP/FLUC in the mBMMs from transgenic FVB mice has been reported [14–16]. However, we detected only minimal expression of GFP/FLUC in mBMMs isolated from commercially available transgenic L2G85 BALB/c mouse suggesting that the expression of these reporter genes is dissimilar between transgenic mouse strains. This strain-dependent reporter gene expression along with the time consuming process of backcrossing the GFP/FLUC reporter gene into new mouse strains potentially required to meet the needs of each specific research application limit the usefulness of this otherwise elegant technique. In addition to FLUC transgenic mice, various mice strains with strong GFP expression in their mononuclear cells have been reported in the literature and thus fluorescence based in vivo BLI offers an alternative to the current approach [17, 18]. However, in an attempt to maximize the sensitivity of the reporter cell system for longitudinal in vivo imaging, we chose to develop FLUC based BLI due to the good tissue penetration and high signal-to-noise ratio typically achieved with this technique.

Due to these limitations in currently existing approaches we undertook efforts to develop a practical and reliable method to produce large numbers of GFP/FLUC expressing mouse primary macrophages that could also be applied to macrophages from various mouse strains. We elected to use lentivirus for macrophage transduction due to the relative simplicity and the cost-effectiveness of the virus production process, possibility to produce large quantities of the vector, and the stable, relatively strong gene expression typically achieved after lentivirus mediated transduction. The drawback of this approach is that primary macrophages are highly resistant to the lentivirus mediated gene transfer necessitating the use of additional reagents to facilitate the infection [6]. Both polybrene and DEAE-dextran have been described to facilitate the lentivirus mediated transduction of mouse primary macrophages at the concentrations used in the current study [19]. Using these reagents in conjunction with the lentivirus, we achieved around 15% gene transfer efficiency; a result that we considered suboptimal and sought for alternative approaches. In addition to these reagents, calcineurin inhibitor cyclosporine has been described to facilitate lentivirus mediated transfer to mouse primary cells [6]. Indeed, in agreement with the description by Noser et al. we found that cyclosporine was highly effective and superior to both polybrene and DEAE-dextran in facilitating the lentivirus mediated gene transfer resulting in up to 40% of the macrophage population to express GFP with similar changes observed in FLUC activity after one 24-hour virus infection.

Next we speculated that it might be possible to further enhance the gene transfer by repeating the lentivirus infection. Indeed, we found that repeating the lentivirus infection after a 24-hour rest period resulted up to 60% of the macrophage population expressing GFP with closely correlated increase in the FLUC activity, without loss of cell viability. Interestingly this double infection strategy was effective in the cyclosporine group but not with the polybrene or DEAE-dextran groups in which the number of GFP positive cells remained around 20% both after the single and double infection. The reason for this phenomenon remains unclear.

It seems plausible that the lentivirus infection and/or cyclosporine treatment could alter the macrophage phenotype or activation state e.g. by activating the pattern recognition receptor signaling in the case of the former or via calcineurin inhibition in the case of the latter. To address these questions and to assess the functional properties of lentivirus and cyclosporine transduced reporter macrophage, we evaluated the reporter macrophages ability to assume M1 or M2 phenotypes by treating the cells either with LPS, IFNγ or LPS+IFNγ for various types of M1 macrophage polarization and with IL-4 for M2 polarization. We found that the reporter cells and the non-transduced control macrophages demonstrated nearly identical pattern of M1 (TNF-α, iNOS, IRF5) and M2 (CD206, Arg1, IRF4) marker gene expression in response to these polarization signals. Some relatively minor differences in the expression of individual markers between the control and reporter cells were seen, the most clear cut difference being in the expression of IRF5 between the control and reporter cells. This difference might be partially explained by the technical variation caused by the relatively small upregulation of IRF5 compared to the rest of the marker genes. The baseline expression of M1 and M2 markers between transducted and non-transducted M0 macrophages was similar suggesting that the lentivirus and cyclosporine treatment either did not affect the macrophage polarization or caused only short-lived and transient changes to macrophage phenotype. Indeed, calcineurin and its inhibition has been previously been reported to exert both pro- and anti-inflammatory effects on macrophages [20, 21]. Based on the current results it seems likely that these effects had already ceased by the time our reporter macrophages were polarized 3 days after the removal of lentivirus and cyclosporine.

Next we evaluated how the polarization treatment affected the expression of GFP/FLUC in reporter macrophages. Somewhat unexpectedly the various treatments to induce M1 polarization led into 40% reduction in GFP expression and FLUC activity, while M2 polarization was associated with 30% increased GFP expression and FLUC activity compared to non-polarized M0 macrophages. To elucidate this somewhat unexpected phenomenon we performed transcription factor binding element analysis of the ubiquitin promoter driving the expression of GFP/FLUC to identify M1 and M2 related transcription factors using TFSEARCH [13]. Indeed, we found that the promoter region contained the binding site for CREB, known to be induced by IL-4 signaling, possibly explaining the increased GFP/FLUC expression in M2 macrophages [22]. The reason for down regulation of GFP/FLUC due to LPS or IFNγ treatment remains unclear.

Regardless of the underlying reasons, the dissimilar levels of GFP/FLUC in M0, M1 and M2 macrophages have to be taken into account when comparing the in vivo behavior of these various types of polarized macrophages. One possible but thus far not validated approach would be to normalize the observed in vivo bioluminescence signal to the in vitro determined differences in the GFP/FLUC expression. Also a plasmid construct using a promoter region that would not be affected by the M1 and M2 associated transcription factors could potentially be the solution to this problem.

After injection into the mouse circulation, the M0 reporter macrophages were easily detectable in the lungs immediately after the injection, using bioluminescence imaging. Migration of the labelled reporter macrophages to the site of chronic inflammation at the right distal femur was observed starting at day 2 after the cell injection confirming the in vivo functionality of the reporter cells. Although decreasing steadily, the signal from the lungs and the right distal femur persisted and was clearly detectable up to 20 days post injection indicating reasonably long life span for these cells. Indeed using immunohistochemistry it was possible to detect several aggregations of FLUC positive cells in the histological sections of the inflammatory area of right femur obtained at day 21 post-injection. Corresponding to the BLI results only few FLUC positive cells were seen in the left control femurs. Similar homing of FLUC expressing RAW 264.7 cells to the area of inflammation and osteolysis in the same mouse model has been previously described [11, 12].

It is to be noted that the mouse model system of biomaterial wear debris-induced inflammation is somewhat unique. Considering the generic nature of biomaterial debris-induced low-grade chronic inflammation [3, 23] we, however, believe that the model adequately serves as a proof of principle of the functionality of the reporter macrophages and that results obtained would similarly reflect the behavior of the reporter macrophages in other disease model systems. Indeed, the in vivo behavior of M0, M1 and M2 macrophages in this model and in other mice models of inflammation, neoplasia, and tissue regeneration will be an intriguing topic for further studies.

In conclusion, large numbers of GFP and FLUC expressing reporter macrophages can be easily and cost-effectively produced using a lentivirus vector and cyclosporine. Transduced reporter macrophages maintain their ability to assume different phenotypes but the GFP/FLUC expression is altered by the polarizing signals. These reporter cells could prove valuable tools to study the role of macrophages in health and disease.

## Supporting Information

S1 Table Transcription factor binding sites at the ubiquitin promoter driving the expression of GFP/FLUC as identified by TFSEARCH. (DOCX)
