# Supplementary material for: Establishment of Green Fluorescent Protein and Firefly Luciferase Expressing Mouse Primary Macrophages for In Vivo Bioluminescence Imaging
Source: PLoS One. 2015 Nov 10;10(11):e0142736. doi: 10.1371/journal.pone.0142736 (PMC4640705; doi:10.1371/journal.pone.0142736)
Supplement: S1 Table — (DOCX) [file pone.0142736.s001.docx]

**TFSEARCH Search Result**

** TFSEARCH ver.1.3 ** (c)1995 Yutaka Akiyama (Kyoto Univ.)

This simple routine searches highly correlated sequence fragments

versus TFMATRIX transcription factor binding site profile database

by E.Wingender, R.Knueppel, P.Dietze, H.Karas (GBF-Braunschweig).

<Warning> Scoring scheme is so straightforward in this version.

score = 100.0 * ('weighted sum' - min) / (max - min)

The score does not properly reflect statistical significance!

Database: TRANSFAC MATRIX TABLE, Rel.3.3 06-01-1998

Query: Lentivirus promoter (1236 bases)

Taxonomy: Vertebrate

Threshold: 85.0 point

TFMATRIX entries with High-scoring:

1 AACCCGTGTC GGCTCCAGAT CTGGCCTCCG CGCCGGGTTT TGGCGCCTCC entry score

--------> [M00050](http://www.cbrc.jp/htbin/bget_tfmatrix?M00050) E2F 100.0

----------> [M00075](http://www.cbrc.jp/htbin/bget_tfmatrix?M00075) GATA-1 85.3

<---------- [M00075](http://www.cbrc.jp/htbin/bget_tfmatrix?M00075) GATA-1 85.3

51 CGCGGGCGCC CCCCTCCTCA CGGCGAGCGC TGCCACGTCA GACGAAGGGC entry score

<------- [M00039](http://www.cbrc.jp/htbin/bget_tfmatrix?M00039) CREB 86.1

101 GCAGCGAGCG TCCTGATCCT TCCGCCCGGA CGCTCAGGAC AGCGGCCCGC entry score

<---------- [M00147](http://www.cbrc.jp/htbin/bget_tfmatrix?M00147) HSF2 86.5

----------> [M00075](http://www.cbrc.jp/htbin/bget_tfmatrix?M00075) GATA-1 86.5

151 TGCTCATAAG ACTCGGCCTT AGAACCCCAG TATCAGCAGA AGGACATTTT entry score

<---------- [M00075](http://www.cbrc.jp/htbin/bget_tfmatrix?M00075) GATA-1 92.2

<---------- [M00076](http://www.cbrc.jp/htbin/bget_tfmatrix?M00076) GATA-2 90.1

<----- [M00271](http://www.cbrc.jp/htbin/bget_tfmatrix?M00271) AML-1a 87.4

201 AGGACGGGAC TTGGGTGACT CTAGGGCACT GGTTTTCTTT CCAGAGAGCG entry score

<------ [M00148](http://www.cbrc.jp/htbin/bget_tfmatrix?M00148) SRY 90.0

251 GAACAGGCGA GGAAAAGTAG TCCCTTCTCG GCGATTCTGC GGAGGGATCT entry score

----------> [M00075](http://www.cbrc.jp/htbin/bget_tfmatrix?M00075) GATA-1 89.8

301 CCGTGGGGCG GTGAACGCCG ATGATTATAT AAGGACGCGC CGGGTGTGGC entry score

---------------> [M00252](http://www.cbrc.jp/htbin/bget_tfmatrix?M00252) TATA 87.7

----------> [M00008](http://www.cbrc.jp/htbin/bget_tfmatrix?M00008) Sp1 87.7

----------> [M00075](http://www.cbrc.jp/htbin/bget_tfmatrix?M00075) GATA-1 86.5

------> [M00101](http://www.cbrc.jp/htbin/bget_tfmatrix?M00101) CdxA 86.4

351 ACAGCTAGTT CCGTCGCAGC CGGGATTTGG GTCGCGGTTC TTGTTTGTGG entry score

<------- [M00148](http://www.cbrc.jp/htbin/bget_tfmatrix?M00148) SRY 90.9

---------> [M00075](http://www.cbrc.jp/htbin/bget_tfmatrix?M00075) GATA-1 88.2

<----------- [M00072](http://www.cbrc.jp/htbin/bget_tfmatrix?M00072) CP2 87.5

401 ATCGCTGTGA TCGTCACTTG GTGAGTAGCG GGCTGCTGGG CTGGCCGGGG entry score

<------- [M00240](http://www.cbrc.jp/htbin/bget_tfmatrix?M00240) Nkx-2. 88.4

---- [M00053](http://www.cbrc.jp/htbin/bget_tfmatrix?M00053) c-Rel 86.0

451 CTTTCGTGGC CGCCGGGCCG CTCGGTGGGA CGGAAGCGTG TGGAGAGACC entry score

-----> [M00053](http://www.cbrc.jp/htbin/bget_tfmatrix?M00053) c-Rel 86.0

501 GCCAAGGGCT GTAGTCTGGG TCCGCGAGCA AGGTTGCCCT GAACTGGGGG entry score

551 TTGGGGGGAG CGCACAAAAT GGCGGCTGTT CCCGAGTCTT GAATGGAAGA entry score

-------> [M00083](http://www.cbrc.jp/htbin/bget_tfmatrix?M00083) MZF1 92.2

<----------- [M00072](http://www.cbrc.jp/htbin/bget_tfmatrix?M00072) CP2 87.5

601 CGCTTGTGAG GCGGGCTGTG AGGTCGTTGA AACAAGGTGG GGGGCATGGT entry score

-------> [M00148](http://www.cbrc.jp/htbin/bget_tfmatrix?M00148) SRY 90.9

---------> [M00008](http://www.cbrc.jp/htbin/bget_tfmatrix?M00008) Sp1 90.4

----------> [M00008](http://www.cbrc.jp/htbin/bget_tfmatrix?M00008) Sp1 89.0

------> [M00271](http://www.cbrc.jp/htbin/bget_tfmatrix?M00271) AML-1a 88.7

<----------- [M00073](http://www.cbrc.jp/htbin/bget_tfmatrix?M00073) deltaE 85.2

651 GGGCGGCAAG AACCCAAGGT CTTGAGGCCT TCGCTAATGC GGGAAAGCTC entry score

<-------- [M00050](http://www.cbrc.jp/htbin/bget_tfmatrix?M00050) E2F 90.8

------------> [M00087](http://www.cbrc.jp/htbin/bget_tfmatrix?M00087) Ik-2 87.3

--------- [M00147](http://www.cbrc.jp/htbin/bget_tfmatrix?M00147) HSF2 85.3

701 TTATTCGGGT GAGATGGGCT GGGGCACCAT CTGGGGACCC CTGACGTGAA entry score

<---------- [M00075](http://www.cbrc.jp/htbin/bget_tfmatrix?M00075) GATA-1 94.7

-------> [M00041](http://www.cbrc.jp/htbin/bget_tfmatrix?M00041) CRE-BP 94.6

-------> [M00039](http://www.cbrc.jp/htbin/bget_tfmatrix?M00039) CREB 92.6

<---------- [M00076](http://www.cbrc.jp/htbin/bget_tfmatrix?M00076) GATA-2 89.3

--------> [M00083](http://www.cbrc.jp/htbin/bget_tfmatrix?M00083) MZF1 87.8

----------> [M00075](http://www.cbrc.jp/htbin/bget_tfmatrix?M00075) GATA-1 87.8

<---------------- [M00066](http://www.cbrc.jp/htbin/bget_tfmatrix?M00066) Tal-1a 86.9

<------- [M00041](http://www.cbrc.jp/htbin/bget_tfmatrix?M00041) CRE-BP 86.9

----------> [M00076](http://www.cbrc.jp/htbin/bget_tfmatrix?M00076) GATA-2 86.6

<------- [M00039](http://www.cbrc.jp/htbin/bget_tfmatrix?M00039) CREB 86.1

<--------- [M00077](http://www.cbrc.jp/htbin/bget_tfmatrix?M00077) GATA-3 85.9

-----------------> [M00059](http://www.cbrc.jp/htbin/bget_tfmatrix?M00059) YY1 85.7

> [M00147](http://www.cbrc.jp/htbin/bget_tfmatrix?M00147) HSF2 85.3

751 GTTTGTCACT GACTGGAGAA ACTCGGGTTT GTCGTCTGTT GCGGGGGCGG entry score

------------> [M00243](http://www.cbrc.jp/htbin/bget_tfmatrix?M00243) Egr-1 91.2

------------> [M00244](http://www.cbrc.jp/htbin/bget_tfmatrix?M00244) NGFI-C 90.3

------------> [M00246](http://www.cbrc.jp/htbin/bget_tfmatrix?M00246) Egr-2 89.3

------------> [M00245](http://www.cbrc.jp/htbin/bget_tfmatrix?M00245) Egr-3 85.4

801 CAGTTATGGC GGTGCCGTTG GGCAGTGCAC CCGTACCTTT GGGAGCGCGC entry score

<--------- [M00227](http://www.cbrc.jp/htbin/bget_tfmatrix?M00227) v-Myb 96.8

---------> [M00141](http://www.cbrc.jp/htbin/bget_tfmatrix?M00141) Lyf-1 92.2

<----------- [M00072](http://www.cbrc.jp/htbin/bget_tfmatrix?M00072) CP2 89.6

------> [M00101](http://www.cbrc.jp/htbin/bget_tfmatrix?M00101) CdxA 87.9

------------> [M00087](http://www.cbrc.jp/htbin/bget_tfmatrix?M00087) Ik-2 87.7

851 GCCCTCGTCG TGTCGTGACG TCACCCGTTC TGTTGGCTTA TAATGCAGGG entry score

--------> [M00041](http://www.cbrc.jp/htbin/bget_tfmatrix?M00041) CRE-BP 100.0

--------> [M00039](http://www.cbrc.jp/htbin/bget_tfmatrix?M00039) CREB 100.0

<-------- [M00041](http://www.cbrc.jp/htbin/bget_tfmatrix?M00041) CRE-BP 100.0

<-------- [M00039](http://www.cbrc.jp/htbin/bget_tfmatrix?M00039) CREB 100.0

<------------ [M00113](http://www.cbrc.jp/htbin/bget_tfmatrix?M00113) CREB 99.2

-------> [M00101](http://www.cbrc.jp/htbin/bget_tfmatrix?M00101) CdxA 90.7

------------> [M00113](http://www.cbrc.jp/htbin/bget_tfmatrix?M00113) CREB 90.2

<------- [M00101](http://www.cbrc.jp/htbin/bget_tfmatrix?M00101) CdxA 86.4

------------> [M00131](http://www.cbrc.jp/htbin/bget_tfmatrix?M00131) HNF-3b 85.5

901 TGGGGCCACC TGCCGGTAGG TGTGCGGTAG GCTTTTCTCC GTCGCAGGAC entry score

<--------------- [M00002](http://www.cbrc.jp/htbin/bget_tfmatrix?M00002) E47 97.1

-----> [M00271](http://www.cbrc.jp/htbin/bget_tfmatrix?M00271) AML-1a 92.0

--------> [M00217](http://www.cbrc.jp/htbin/bget_tfmatrix?M00217) USF 89.4

---------------> [M00002](http://www.cbrc.jp/htbin/bget_tfmatrix?M00002) E47 86.5

<------------ [M00001](http://www.cbrc.jp/htbin/bget_tfmatrix?M00001) MyoD 86.0

951 GCAGGGTTCG GGCCTAGGGT AGGCTCTCCT GAATCGACAG GCGCCGGACC entry score

1001 TCTGGTGAGG GGAGGGATAA GTGAGGCGTC AGTTTCTTTG GTCGGTTTTA entry score

----- [M00101](http://www.cbrc.jp/htbin/bget_tfmatrix?M00101) CdxA 92.1

----- [M00100](http://www.cbrc.jp/htbin/bget_tfmatrix?M00100) CdxA 91.0

--------> [M00083](http://www.cbrc.jp/htbin/bget_tfmatrix?M00083) MZF1 90.4

<------ [M00148](http://www.cbrc.jp/htbin/bget_tfmatrix?M00148) SRY 90.0

-------> [M00240](http://www.cbrc.jp/htbin/bget_tfmatrix?M00240) Nkx-2. 88.4

--------------> [M00127](http://www.cbrc.jp/htbin/bget_tfmatrix?M00127) GATA-1 88.0

-----------> [M00203](http://www.cbrc.jp/htbin/bget_tfmatrix?M00203) GATA-X 86.6

1051 TGTACCTATC TTCTTAAGTA GCTGAAGCTC CGGTTTTGAA CTATGCGCTC entry score

<---------- [M00076](http://www.cbrc.jp/htbin/bget_tfmatrix?M00076) GATA-2 92.9

-> [M00101](http://www.cbrc.jp/htbin/bget_tfmatrix?M00101) CdxA 92.1

<---------- [M00075](http://www.cbrc.jp/htbin/bget_tfmatrix?M00075) GATA-1 91.4

-> [M00100](http://www.cbrc.jp/htbin/bget_tfmatrix?M00100) CdxA 91.0

<----------- [M00203](http://www.cbrc.jp/htbin/bget_tfmatrix?M00203) GATA-X 89.8

<--------- [M00077](http://www.cbrc.jp/htbin/bget_tfmatrix?M00077) GATA-3 89.1

<------------- [M00128](http://www.cbrc.jp/htbin/bget_tfmatrix?M00128) GATA-1 88.4

------> [M00240](http://www.cbrc.jp/htbin/bget_tfmatrix?M00240) Nkx-2. 86.0

1101 GGGGTTGGCG AGTGTGTTTT GTGAAGTTTT TTAGGCACCT TTTGAAATGT entry score

<------ [M00148](http://www.cbrc.jp/htbin/bget_tfmatrix?M00148) SRY 92.7

--------------> [M00109](http://www.cbrc.jp/htbin/bget_tfmatrix?M00109) C/EBPb 91.8

----- [M00137](http://www.cbrc.jp/htbin/bget_tfmatrix?M00137) Oct-1 88.4

1151 AATCATTTGG GTCAATATGT AATTTTCAGT GTTAGACTAG TAAATTGTCC entry score

<-------------- [M00162](http://www.cbrc.jp/htbin/bget_tfmatrix?M00162) Oct-1 91.8

-------> [M00137](http://www.cbrc.jp/htbin/bget_tfmatrix?M00137) Oct-1 88.4

<------- [M00101](http://www.cbrc.jp/htbin/bget_tfmatrix?M00101) CdxA 87.1

<------- [M00101](http://www.cbrc.jp/htbin/bget_tfmatrix?M00101) CdxA 86.4

------------> [M00045](http://www.cbrc.jp/htbin/bget_tfmatrix?M00045) E4BP4 85.3

1201 GCTAAATTCT GGCCGTTTTT GGCTTTTTTG TTAGAC entry score

-------> [M00101](http://www.cbrc.jp/htbin/bget_tfmatrix?M00101) CdxA 92.1

<------- [M00148](http://www.cbrc.jp/htbin/bget_tfmatrix?M00148) SRY 86.4

<------------ [M00160](http://www.cbrc.jp/htbin/bget_tfmatrix?M00160) SRY 85.9

Total 88 high-scoring sites found.

Max score: 100.0 point, Min score: 85.2 point
